# Inpatient Management of Pain Episodes in Children with Sickle Cell Disease: A Review

**DOI:** 10.3390/children11091106

**Published:** 2024-09-10

**Authors:** Zhour Barnawi, Ronay Thomas, Radhika Peddinti, Nabil Abou Baker

**Affiliations:** 1 Department of Pediatrics, Section of Hematology-Oncology and Bone Marrow Transplant, University of Chicago Medicine, Chicago, IL 60637, USA; zhour.barnawi@uchicagomedicine.org (Z.B.); rpeddint@bsd.uchicago.edu (R.P.); 2 Department of Pediatrics, King Abdulaziz University, Jeddah 21589, Saudi Arabia; 3 Department of Medicine and Department of Pediatrics, University of Chicago, Chicago, IL 60637, USA; ronay.thomas@uchicagomedicine.org

**Keywords:** sickle cell disease, vaso-occlusive pain, vaso-occlusive event, vaso-occlusive episode, vaso-occlusive crisis, children with sickle cell disease, inpatient management of pain

## Abstract

Sickle cell disease (SCD) is the most common hemoglobinopathy in the world. Sickle cell vaso-occlusive episodes (VOEs) are very painful acute events and the most common complication as well as reason for hospitalization. SCD pain is best evaluated holistically with a pain functional assessment to aid in focusing pain management on reducing pain in addition to improving function. Patients with SCD have long endured structural racism and negative implicit bias surrounding the management of pain. Thus, it is important to approach the management of inpatient pain systematically with the use of multi-modal medications and nonpharmacologic treatments. Furthermore, equitable pain management care can be better achieved with standardized pain plans for an entire system and individualized pain plans for patients who fall outside the scope of the standardized pain plans. In this article, we discuss the best practices to manage SCD VOEs during an inpatient hospitalization.

## 1. Introduction

Sickle cell disease (SCD) is the most prevalent hemoglobinopathy worldwide, affecting 70,000 to 100,000 individuals in the United States, with Blacks being disproportionately affected. Approximately 1 of 356 African American/Black births result in an infant with SCD [1,2]. SCD encompasses different disease genotypes, including sickle cell anemia (HbSS), hemoglobin SC disease, and hemoglobin S beta-thalassemia [3]. HbSS is inherited in an autosomal recessive pattern and caused by a missense point mutation resulting in the replacement of glutamine with valine at the sixth position of the beta globin gene in chromosome 11 [4]. This mutation alters normal hemoglobin folding, leading to the formation of a defective, hydrophobic hemoglobin called hemoglobin S “HbS”. This form of hemoglobin results in fragile, sickle-shaped red blood cells that are prone to HbS polymerization whenever they encounter deoxygenation. These sickle-shaped cells make travel through narrow blood vessels more difficult, leading to cell lysis while simultaneously triggering numerous molecular and cellular processes including endothelial injury, abnormal cell aggregation, inflammation activation, and adhesion to blood vessel walls, leading to ischemic reperfusion damage [4,5,6].

SCD is a complex and multi-systemic disease leading to a wide range of acute and chronic complications that can be broadly characterized as vaso-occlusive episodes (VOEs), complications of hemolysis, and sequelae of chronic inflammation [1,7]. The most common of these complications is pain episodes related to vaso-occlusive episodes. This review article focuses on summarizing the current published research and knowledge related to different types of SCD-related pain and the most recent principles regarding inpatient management of SCD pain among pediatric populations. There is a lack of a unified national protocol for managing SCD-related pain at this time; hence, this review aimed to provide comprehensive clinical guidance for pediatricians to utilize for managing pain related to SCD.

## 2. Overview of SCD-Related Pain and Risk Factors

Pain episodes are a hallmark complication of SCD and the most common reason for seeking medical attention and hospitalization among children and adults with SCD [7,8,9]. The incidence of the first VOE increases with the child’s age with 6% of children developing their first VOE by six months of age and 96% of children by eight years of age [10]. Pain episodes range in duration, severity, and location. In children, the severe, self-limiting pain is often a direct result of discrete occlusive events and tissue ischemia, however, as these patients age, the mechanism becomes more complex with diffuse inflammation, underlying chronic pain, and hemolysis among the contributing factors. Over time, the pain character might evolve to become more chronic [11]. In addition, the increasing frequency and severity of acute vaso-occlusive pain episodes in adolescence and adulthood are associated with an increased mortality rate [12,13,14]. Nonetheless, these recurrent pain episodes negatively affect the quality of life of individuals with SCD and increase healthcare utilization [15,16,17].

**Figure 1:**
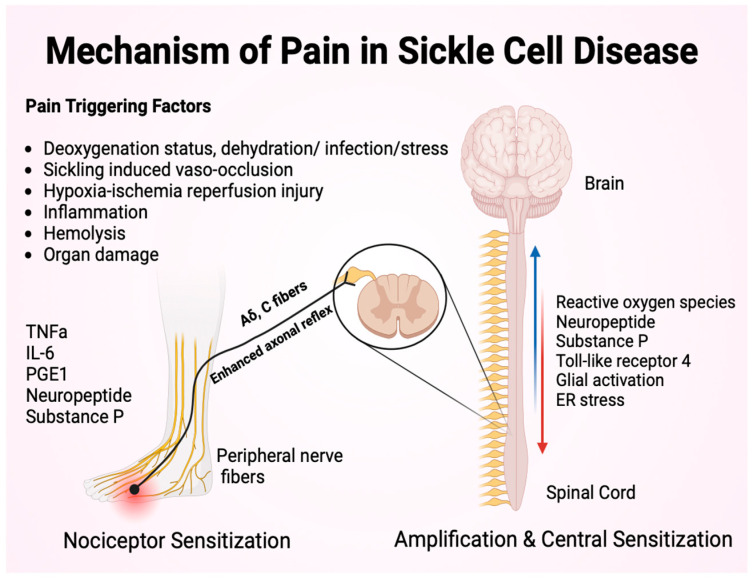
This figure shows the pain’s pathway from the peripheral nerve to the brain. During moments of factors that trigger pain, mast cells activate and release neuropeptide substance P. There are increased cytokines such as tumor necrosis factor-alpha (TNFα), interleukin 6 (IL-6), and prostaglandin E1 (PGE1). In turn, this initiates pain signaling down the dorsal root ganglion and spinal cord to the brain. In central sensitization, there is increased reactive oxygen species, endoplasmic reticulum (ER) stress, glial activation, toll-like receptor 4 phosphorylation of p38MAPK.

Factors triggering acute pain episodes include viral illness, surgery, stress, extreme weather changes, strenuous exercise, dehydration, and any status leading to poor oxygenation [18,19]. In some cases, a specific trigger may not be identified. These factors trigger the process of peripheral and central sensitization to pain, as illustrated in Figure 1. The pain pathway from a localized site of pain, transmitted through peripheral nerve fibers and reaching the pain’s center in the brain, is also shown in Figure 1 [20].

A holistic evaluation of the patient may yield other factors that are contributing to the pain. Using the biopsychosocial framework in sickle cell disease identifies additional contributors to the pain. For example, when evaluating, the psychological evaluation may yield a mental health disorder such as depression, anxiety, or a sleep disorder, and a sociological evaluation may identify stigma, environmental stressors, trauma, and disparities [21].

## 3. Classification of Pain in SCD

**Figure 2:**
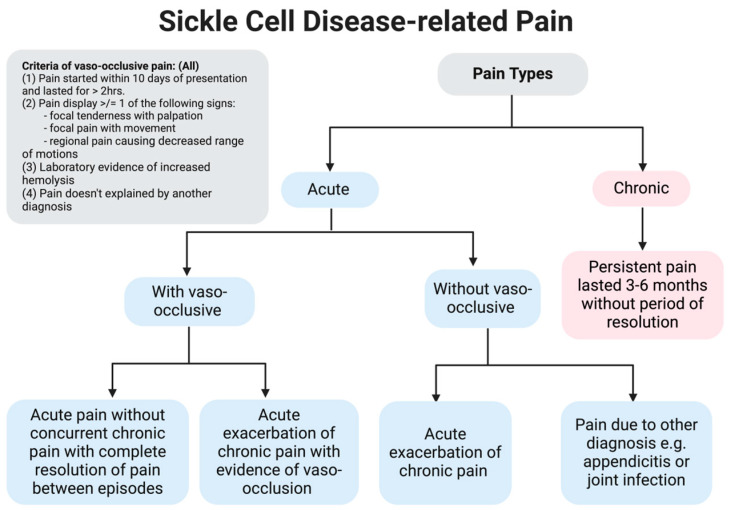
This flow chart shows the different types of pain related to SCD. There are four main types of acute pain: acute VOE pain without chronic pain between VOEs, acute VOE pain with chronic pain in between VOEs, acute on chronic pain exacerbation without VOE, and pain due to another diagnosis. It is important to evaluate the pain and ensure that a VOE pain mimicker such as infection is not the cause as management may change.

Pain in the setting of SCD primarily can be classified into two major types depending on acuity, as shown in Figure 2: acute pain which starts within a few days of presentation and chronic pain which persists within months to years of symptom onset. Recurrent acute pain may be differentiated from chronic pain in that there is no period of complete resolution of symptoms for patients with chronic pain [22]. As defined by the AAAPT, acute pain due to a VOE must meet the following criteria: confirmed diagnosis of SCD via laboratory testing, increased pain lasting greater than two hours and starting within 10 days of presentation, pain not ascribed to a specific exam finding or imaging abnormality, and finally must be reproducible with palpation, movement, or with noted decreased motion of the area [11]. In addition, pain due to VOEs can occur with or without concurrent chronic SCD pain.

In children, these episodes typically occur without evidence of chronic pain, meaning symptoms may fully resolve between episodes. VOEs may also present with laboratory findings including hemoglobin levels below the patient’s baseline, an increased reticulocyte count, and elevated lactate dehydrogenase (LDH). Acute pain that occurs in the setting of chronic SCD pain may present with or without the physical or hematologic signs of VOEs listed above. Currently, work is underway to evaluate if genetic variants may contribute to the pain phenotype. When evaluating single nucleotide polymorphisms (SNPs) in patients with SCD, several are associated with acute pain, while a few are associated with both acute and chronic or only chronic pain [23].

## 4. Common Features and Assessment of Pain

Children may begin to experience VOEs as early as 6 months of age as fetal hemoglobin begins to decline [10]. Although the characterization of pain can be challenging in younger children, associated symptoms include tachycardia, fever, and a decreased range of motion in the affected area. Dactylitis associated with pain and swelling of the hands and feet tends to occur in infancy, however, the spine, pelvis, and long and flat bones are the common pain sites for older patients [24]. Researchers have described acute pain episodes as having four distinct phases coupled with changes in certain markers of SCD. These phases include the prodromal phase, initial phase, established phase, and resolving phase [25,26,27].

The utilization of age-related pain assessment tools is a crucial step in characterizing VOEs in pediatric patients. Utilizing strictly numeric scales can create inconsistency among providers and patients. For instance, a patient with SCD experiencing a VOE with a 5/10 pain level may still require significant opioid-based analgesia, while a patient without chronic pain experiencing the same level of pain could be managed with nonsteroidal anti-inflammatory drugs (NSAIDs) alone. In addition, younger children may be unable to effectively communicate numeric-based pain scales. As a result, observational–behavioral scales including the Face, Legs, Activity, Cry, and Consolability Scale (FLAAC) and the Children’s Hospital of Eastern Ontario Pain Scale (CHEOPS) are commonly used [28,29]. The FLACC method utilizes facial expression, leg position, activity level, presence or absence of crying, and consolability each on a scale from 0 to 2 to generate an objective pain score from 0 to 10. This assessment tool can be replicated between providers and provides an objective measure that can be followed regardless of the patient’s ability to participate in quantifying pain. This can be especially useful in younger patient populations [29]. Conversely, those with chronic pain who may not exhibit typical features of discomfort may not benefit from assessment tools based solely on physical attributes. Similarly, the CHEOPS scale utilizes behavioral traits to assess pain, although this scale includes additional attributes like the desire to touch/reach and verbal complaints [28]. In younger patients (under age 7), self-evaluation scales, like the Wong–Baker FACES scale that allows children to choose a face representative of their pain level, may be more beneficial [29,30]. Specifically, the FACES scale provides a neutral “no pain” face that allows for more accurate scoring as many other face-based scales utilize a smiling “no pain” face which often results in higher pain ratings [30].

For older patients, using multidimensional patient-reported outcome tools such as ASCQ-ME, which helped measure the impact of pain in a patient’s life, would be the ideal choice [31]. Once an accurate assessment of pain has been obtained, VOEs can be classified as mild, moderate, and severe often based on each patient’s baseline level of pain. A functional pain assessment such as the Youth Acute Pain Functional Ability Questionnaire (YAPFAQ) can be used to assess the physical functional impact of the pain and measure the recovery of function needed to thrive once discharged from the hospital [32]. A functional assessment can be informative in patients with chronic pain who consistently have an elevated numerical scoring [33].

## 5. General Approach for SCD Pain Management

**Figure 3:**
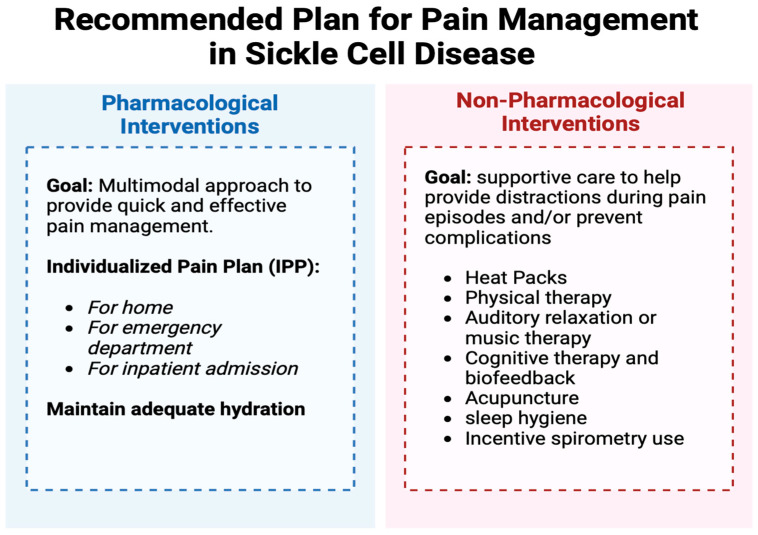
This figure displays different interventions to manage pain related to SCD. In general, there are two large subgroups of interventions: pharmacological and non-pharmacological. It is important that both are used to manage acute pain for the most effective resolution to the pain.

The American Society of Hematology (ASH) strongly encourages the use of individualized pain plans for patients which involve a comprehensive multidisciplinary approach including pharmacological and non-pharmacological interventions as shown in Figure 3 [18,34]. The main clinical goal of pharmacological treatment is to provide rapid and effective pain management. This should be achieved by providing patients with appropriate analgesia and instructing them to start oral pain medications at the onset of symptoms, even if this occurs in the home. Using a multimodal approach that includes a pain regimen with medications targeting different mechanisms to minimize pain perception and inflammation is strongly encouraged [18]. For younger patients, a home regimen would likely include oral Acetaminophen and NSAIDs alternating every 4–6 h with oral opioids given every 6 h for breakthrough pain. Patients are also encouraged to utilize non-pharmacologic pain management strategies at home including warm compresses, massage therapy, and adequate hydration. These interventions are discussed further in later sections. During clinic visits, providers should discuss the signs and symptoms of a VOE as well as acute complications to appropriately triage symptoms at home. For instance, caregivers of patients with SCD should be able to identify hepatosplenomegaly on basic home exams, identify symptoms of ACS (i.e., chest pain, difficulty breathing), and assess if the home pain plan is adequately controlling the patient’s pain. This is a crucial step in ensuring a successful home regime. If home pain management fails, patients should seek medical attention for further evaluation, with additional IV pain medication available if needed.

**Figure 4:**
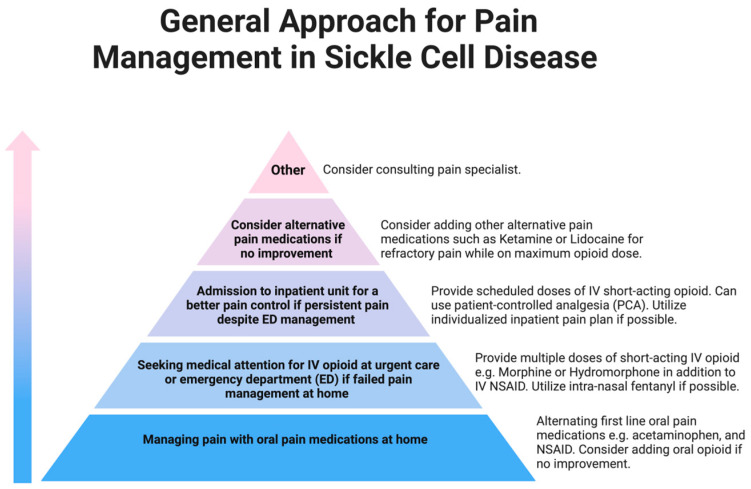
This diagram illustrates the general approach to pharmacological SCD-related pain management. At all levels of the pyramid, patients may utilize non-pharmacological methods to support pain management. At the bottom of the pyramid are inventions that should apply to many patients with SCD while the top of the pyramid is reserved for select patients that require complex management. In the first or bottom level of the pyramid, patients should manage general pain at home with oral medications such as acetaminophen, nonsteroidal anti-inflammatory drugs (NSAIDs), or opioids for severe pain unresponsive to the aforementioned medications. In the 2nd level, the emergency department (ED) may utilize short-acting intravenous (IV) or intranasal (IN) medication to manage the pain. In the 3rd level of the pyramid, patients are admitted to the hospital and should obtain multimodal analgesia for management including the use of a patient-controlled analgesia. In the 4th level during hospitalization, the pain may be refractory to conventional pain medication and need IV ketamine or lidocaine infusion. Of note, if this treatment is highly effective for a patient, then it may be offered with conventional management. On the 5th and final level, additional support from a pain specialist in addition to a multidisciplinary team will need to be considered to manage the pain.

When triaging patients who present with a VOE to the emergency department or urgent care, close monitoring of each patient’s response to initial therapy is required to assess the need for inpatient treatment and escalation of pain management. Figure 4 illustrates the pyramid approach to managing SCD pain and the typical steps taken for escalation of pain management. If the acute pain persists, patients are advised to visit their primary hematologist in clinic or present to an emergency department for escalation of care. In accordance with ASH, patients whose pain is not successfully managed with a home oral pain regimen are typically treated with 2–3 doses of IV opioids in addition to oral or IV NSAIDs [18]. The use of intranasal fentanyl has also been studied in patients with SCD who had been evaluated in the emergency department, and is associated with improved pain relief, decreased need for IV placement, and decreased hospitalization rates [35,36]. Again, if pain persists despite an escalation of care, patients will require inpatient admission to adequately manage the acute pain episode. In addition, those exhibiting signs of other, severe sequelae of SCD listed above should also be admitted for close monitoring and possible additional treatment including blood transfusion and antibiotic therapy [18].

## 6. Inpatient Management of Pain

If possible, the use of a standardized protocol to manage acute pain in hospitalized sickle cell patients is highly recommended. This helps ensure that a comprehensive approach will be taken to provide high-quality and effective pain management. Most hospitals have implemented clinical pathways, such as agile pathways, to achieve this goal in both the emergency department and inpatient units [37,38]. This comprehensive approach should include pharmacologic and non-pharmacologic interventions. Inpatient pain plans should also follow a similar approach to emergency department pain management where multimodal medications are used to effectively treat the pain. The pain regimen should include oral or IV acetaminophen, IV NSAIDs, and intermittent or scheduled short-acting opioids. Pain control might be difficult to achieve with an intermittent dosing of opioids in some patients; hence, escalation is recommended with patient-controlled analgesia (PCA). PCAs provide both continuous (basal) and demand dosing of long-acting pain medications [39]. If minimal or no clinical improvement is observed, alternative medications such as IV ketamine and IV lidocaine can be initiated.

Topical pain medications such as lidocaine patches can also be effective for localized pain [40]. Once the pain has been effectively treated with patient-reported clinical improvement and improvement in pain scores, the gradual de-escalation of pain medication is needed to transition patients to an oral regimen. An opioid calculator should be used when switching between different opioid medications as there can be a 25–50% cross-tolerance requiring dose reduction [41].

Patients should have close clinical and laboratory monitoring while receiving different opioid and non-opioid pain medications to rapidly identify and address any adverse drug effects. Supplementary Materials Table S1 lists the most common pain medications used in the management of SCD-related pain with mechanisms of action, routes of administration, unique clinical features, as well as the side effects of each medication. It is important to note that many medications are metabolized by CYP2D6 and patients with SCD are associated with low enzymatic activity. It is recommended against using codeine, a prodrug for morphine in SCD [42,43].

**Figure 5:**
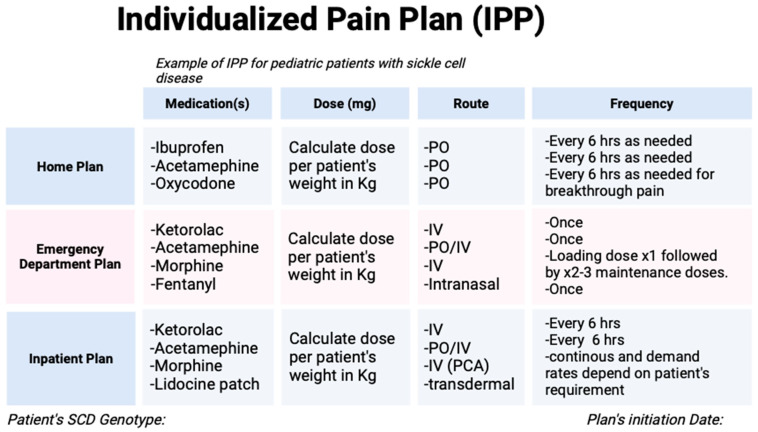
This is an example of an individualized pain plan for a patient with SCD. There should be 3 sections with a plan for home-related pain, emergency room, and inpatient pain plans. Medications should be calculated on weight-based dosing, not predetermined doses. Routes such as oral (PO), intravenous (IV), intranasal, and transdermal should be individually discussed with each patient and their caregivers to ensure understanding and agreement.

Case Presentation: To exemplify the principles of pain management for patients with sickle cell disease, we have included a case presentation and stepwise approach to pain management. A 14-year-old male with sickle cell disease (HbSS) follows at a local hematology office and presents with his parents with a primary complaint of acute onset of right lower abdominal pain for 2 days. He describes the pain as achy in character, progressive, and consistent with previous VOEs. He has no sick contact at home or school. His vitals and physical examination were benign. His hematologist noted evidence of increased hemolysis with a drop in his hemoglobin by 1 g/dL, reticulocytosis, elevated LDH, and mild leukocytosis. He was given a dose of oral oxycodone in clinic and instructed to go home to initiate his individualized pain plan. An example of an individualized pain plan is shown in Figure 5. His home pain plan includes ibuprofen and acetaminophen alternating every 3 h with oxycodone every 6 h for breakthrough pain as needed. Eight hours later, he returned to the emergency department with worsening abdominal pain despite receiving oxycodone within the past two hours. On arrival, he was tachycardic and had two episodes of non-bloody non-bilious emesis. His physical examination was benign. His emergency pain plan was initiated with morphine, acetaminophen, and ketorolac. He received IV fluid for hydration to slow the sickling process. He continued to have persistent abdominal pain despite receiving a total of three doses of morphine which warranted admission.

During hospital admission, a multidisciplinary approach was taken to manage his pain. For pharmacologic therapy, he was started on a morphine PCA with continuous and demand dosing, scheduled IV ketorolac, and scheduled acetaminophen. Supportive care included continuing maintenance of IV fluid and a bowel regimen to prevent the development of constipation. He was monitored clinically for morphine side effects including laboratory monitoring for liver and kidney dysfunction and signs of hemolysis. Additional supportive teams were consulted to provide interventions such as music therapy, physical therapy, massage therapy, and child life interventions.

The patient showed improvement after 48 h of appropriate pain management. His regimen was gradually de-escalated by reducing the morphine PCA demand dosing every few hours. Eventually, his PCA basal dosing was transitioned to an equivalent oral oxycodone dose. He was instructed to wean off his oral pain regimen at home as he continued to improve and to follow up with his primary hematologist within a week of discharge.

## 7. Supportive Care

Although first-line treatment for VOEs is pharmacologic therapy, medication alone does not provide complete pain relief and fails to address the psychological impacts of chronic severe pain [44]. Non-pharmacologic therapies including physical and psychological interventions can be beneficial in minimizing pain and improving quality of life in patients living with SCD. Specifically, physical techniques such as acupuncture and massage therapy directly target muscle spasms and muscle guarding commonly experienced by patients with chronic pain [45]. These methods have been shown to decrease hospital utilization, opioid use, and perceived pain and tension levels. In addition, the application of heat is a relatively benign and accessible method of pain management that can be especially effective in patients with SCD as it causes local vasodilation, leading to increased blood flow and pain relief.

Non-pharmacologic therapies that target the psychological aspects of chronic pain include meditation, music therapy, and appropriate psychiatric care when indicated. The pain management guidelines set by ASH highlight the need for cognitive and behavioral pain management strategies as part of a comprehensive, multidisciplinary pain strategy [18]. Patients with SCD who engage in Cognitive Behavioral Therapy (CBT) often have a reduction in pain intensity, with effects lasting up to six months [46]. Specifically, in pediatric patients with SCD, Cognitive Behavioral Therapy (CBT) and coping skills imagery have been effective in managing acute pain episodes by helping patients identify and address negative emotions associated with the physical pain they experience [18,47]. In addition to reducing rates of hospitalization, these behavioral pain management strategies provide coping strategies that can be used long term [48]. Similarly, music therapy addresses both the physical and psychological aspects of pain by allowing patients to develop distraction techniques and coping skills. The ability to write songs and play instruments provides an outlet for negative feelings related to pain which, in turn, results in decreased pain perception and strengthens disease management skills [49]. 

Other supportive therapies that help manage symptoms related to SCD that can be readily applied in both inpatient and outpatient settings include incentive spirometry (IS), bowel regimens, and hydration therapy. Incentive spirometry encourages lung expansion and improved aeration which can prevent the development of atelectasis. The frequent use of incentive spirometry during hospitalization can reduce the risk of SCD-related complications including acute chest syndrome, pneumonia, and deconditioning. This is especially important in patients receiving opioid-based therapy given the increased risk of sedation and respiratory depression. With the assistance of Child Life Specialists, IS can be easily adapted for use with pediatric patients. Similarly, opioid use can lead to significant constipation and bowel discomfort, leading to significant pain and morbidity when left untreated. Therefore, the introduction of a bowel regimen and appropriate hydration with monitoring for daily voiding is crucial in maintaining gut health and improving the quality of life in patients with SCD [50].

Although non-pharmacologic measures can be effective pain management strategies, they are inconsistently used in the inpatient setting, especially in the adolescent and young adult population who are eligible for child life interventions. In addition, most data on non-pharmacologic interventions in patients with SCD focus on adult populations, which highlights the need for further evaluation of alternative pain management in pediatric patients.

## 8. Preventative Treatment

One of the most effective ways to manage a patient’s pain is working to improve or prevent future episodes. Non-pharmacologic strategies to manage health should be utilized by all patients such as eating a balanced diet and obtaining regular physical activity. However, in patients with sickle cell disease, it is vital to ensure monitoring of triggers and avoidance if possible. Trigger avoidance is best when paired with patient and caregiver education to observe for these triggers and create home plans that may address a common specific issue affecting a patient.

In the last several years, new disease-modifying treatments have been approved for use by the Federal Drug Administration (FDA). Today, there are four main disease-modifying drugs: hydroxyurea, L-Glutamine, Voxelator, and Crizanlizumab [51]. It is important that patients are offered preventative treatment if they are having recurrent VOEs, especially requiring hospitalization. Hydroxyurea has been shown to decrease VOEs by 44%, increase the time interval between VOEs, and decrease acute chest syndrome and transfusions [52]. L-Glutamine, the second drug to be approved by the FDA for SCD, functions by reducing oxidative stress, which decreases VOEs by 25% and decreases hospital admissions by 33% [53]. Voxelotor is a hemoglobin polymerization inhibitor that increases the mean hemoglobin by 1 g/dL and subjectively decreases pain severity in patients [54,55]. Crizanlizumab is a monoclonal antibody that binds P-selectin to inhibit the inflammatory cascade and reduces VOEs by 45–63% [56].

In addition to disease-modifying treatments, curative therapies have been on the rise. In 2023, gene therapy was approved by the FDA, offering patients with SCD an alternative to the allogeneic hematopoietic cell transplant. These patients do have fewer pain episodes directly related to VOEs and improvement in chronic pain [57].

## 9. Special Considerations

We aim to underscore the key considerations for patients with sickle cell disease that pediatricians must acknowledge. Children and adults with SCD have been impacted by structural racism, healthcare disparities, and negative stigma within the healthcare system, especially in the United States of America [58,59,60]. Many patients with SCD have been subjected to direct and indirect harm as a result of healthcare legislation, lack of SCD research funding, inadequate access to healthcare, suboptimal symptom management, and implicit or explicit biases expressed by healthcare providers [59]. The complexity of SCD and its sequelae, in particular acute vaso-occlusive pain episodes, requires frequent visits to healthcare facilities for pain management [13]. As mentioned in this review, opioid medications and other controlled substances are considered the cornerstone of acute and chronic SCD-related pain management, however, there has been a significant deficiency in creating standardized protocols to manage pain for many years. This, unfortunately, has led to the marginalization of patients with SCD and delays in providing high-quality care. In addition, the need for frequent visits to hospitals for pain management has led to improper labeling of patients, which ultimately leads to inadequate pain management. Such behaviors have been deeply ingrained within the healthcare system, resulting in growing patient mistrust and an increased prevalence of self-doubt, depression, anxiety, and a negative self-image among patients with SCD [61].

Moreover, the perception of pain is inherently subjective. Clinicians are encouraged to rely on patient-reported pain, assess patients’ functionality, and use a validated pain assessment tool for younger patients to address their pain fairly. It is important to acknowledge that vital signs changes and laboratory evidence of hemolysis are not always observed during pain episodes. Pediatricians should be aware of these special considerations to properly advocate for patients with SCD.

## 10. Conclusions

- Acute SCD-related pain is the most common complication and requires fast and effective multimodal pain management and vigorous hydration.
- It is highly recommended to have an individualized pain plan for home, emergency department visit, and inpatient given the variability of a patient’s response to pain medications.
- Pediatricians should have a broad differential diagnosis of pain in patients with SCD to avoid misdiagnosis and delaying therapeutic interventions for other infectious and noninfectious causes of pain which might present similar to vaso-occlusive pain episodes.
- Clinicians should be aware of the impact of structural racism, healthcare disparities, and negative stigma on patients with SCD and advocate to implement changes.

## Supplementary Materials

The following supporting information can be downloaded at: https://www.mdpi.com/article/10.3390/children11091106/s1, Table S1: Common medication used for treating sickle cell disease-related pain.
