# Supplementary material for: Inpatient Management of Pain Episodes in Children with Sickle Cell Disease: A Review
Source: Children (Basel). 2024 Sep 10;11(9):1106. doi: 10.3390/children11091106 (PMC11430294; doi:10.3390/children11091106)
Supplement: Supplementary file 1 [file children-11-01106-s001.zip › children-3150600-supplementary.pdf]

Supplementary Materials

Table S1: Common medications used for treating sickle cell disease-related pain.

Type of medications	Class of Medication, Route	Mechanism of Action	Unique Features & Clinical Utilization
Non-Opioid			
	Acetaminophen (PO/IV)	Inhibits prostaglandins biosynthesis in the brain by blocking cyclooxygenase. This leads to decrease pain.	-Used with caution if patient is taking a combined drug such as hydrocode-acetaminophen to avoid drug overdose and subsequent liver injury.
	Non-steroidal anti-inflammatory drugs (e.g., Ibuprofen (PO), Ketorolac (IV), etc)	Non-selective cyclooxygenase inhibitors. Decreasing the biosynthesis of prostaglandins in the whole-body results in decreased pain and inflammation.	-Ketorolac considered to be a standard of care in treating Vaso-occlusive pain management given it's anti-inflammatory effect.<br>-Some recommendation limited it's use to 5-day given the risk of GI bleeding.<br>-Used with caution in patients with renal impairment.
	Gabapentinoids (e.g., Gabapentin PO)	Binds to the alpha-2-delta-1 subunits which leads to block Ca+ channel in central nervous system. This reduces neuronal excitability and blocks the development of hyperalgesia and central sensitization.	-Used mainly for chronic neuropathic pain, and has no utility for acute pain management
	Serotonin and norepinephrine reuptake inhibitors (SNRI)	Inhibits serotonin and norepinephrine reuptake leading to enhanced descending inhibition of centrally sensitized pain. Accumulation of neurotransmitter blocks the transmission of pain signals. Used for neuropathic and chronic pain.	-Major class of antidepressants and anxiety.
Opioid			
A-Strong (Full) agonists bind to mu opioid receptor avidly on central & peripheral nervous system and provide maximal activation to decrease pain			
Short acting (4-6 hrs)	Morphine (PO/IV)- (3-4 hrs).<br>**It also called opiate; naturally occurring alkaloid derived from opium**	-Decreasing pre-synaptic release of excitatory neurotransmitters<br>-Decreasing post-synaptic neuronal excitability<br>-Promoting descending inhibition	-Used for acute pain management<br>-Side effects include respiratory depression, bradycardia, hallucination, euphoria, tolerance/dependence, decrease GI motility and itching as opioid receptors distributed throughout the brain, spinal cord, peripheral nervous system, skin, and joints.<br>-Used with caution in patients with renal impairment.
	Hydromorphone (PO/IV) (3-4 hrs)<br>**Semisynthetic**	Similar to Morphine	-Contraindication in the setting of known allergies to hydromorphone

			-Similar side effect to Morphine
	Oxycodone (PO) (3-4 IR, 8-12 CR)<br>**Semisynthetic**	Agonist to mu, kappa, and delta receptors	-Similar side effect to Morphine
	Fentanyl (IV for PCA) and (IN for emergency pain control)<br>**Synthetic**	Binds to mu receptors and decrease pain neurotransmitters.	-Required liver function monitoring<br>-Intranasal fentanyl recommended to use as part of IPP for the emergency setting for quicker pain control in the setting of VOE. It shows to decrease the need for IV-line placement. Some patients might experience local irritation
Long acting (6-12 hrs)	Methadone (PO) (6-8 hrs)<br>**Synthetic**	Mu receptor agonist, NMDA receptor antagonist and serotonin and norepinephrine uptake inhibitor. Decreased pain	--Required liver function monitoring<br>-Indicated for patients with opioid tolerance, opioid induced hyperalgesia/neurotoxicity, morphine allergy or renal failure/on dialysis
B-Weak against binds to opioid receptors to decrease pain			
	Hydrocodone (3-4hrs)<br>**Semisynthetic**	Binds to mu receptors to produce analgesic effects in both the brain and spinal cord.	-Side effects including respiratory depression, sedation, dependance, constipation and urinary retention.
	Codeine (PO) (4-6hrs)<br>**Naturally occurring alkaloid derived from opium**	Binds to mu and delta receptors as a weak agonist to produce analgia effects.	-In USA, it is contraindications in patients <12 years and patients <18 years following tonsillectomy given the risk for respiratory depression and death.
C-Partial agonists binds to opioid receptors on central & peripheral nervous system and provide sub-maximal effects to decrease pain			
	Buprenorphine (PO)<br>**Semisynthetic**	Acts as a partial agonist on mu opioid receptor and antagonist of the kappa and delta opioid receptors.	-approved to treat both chronic pain and opioid use disorder
Alternative medications			
	Ketamine (IV)	Rapid acting noncompetitive NMDA receptors antagonist	-Required liver function monitoring<br>-Reported evidence of significant reduction in opioid consumption and tolerance in patients used Ketamine for VOE.<br>-Low-dose can be used for opioid-induced hyperalgesia
	Lidocaine (Transdermal/IV)	Blocking peripheral and central voltage-gated Na+ channels leading to a reversible block of action potential propagation and reduced nerve signaling.	-Systemic route needs close monitoring given risk for bradycardia, hypotension, arrythmia, seizure and coma.
